# Impaired learning to dissociate advantageous and disadvantageous risky choices in adolescents

**DOI:** 10.1038/s41598-022-10100-7

**Published:** 2022-04-20

**Authors:** Marieke Jepma, Jessica V. Schaaf, Ingmar Visser, Hilde M. Huizenga

**Affiliations:** Department of Psychology, University of Amsterdam, Amsterdam, the Netherlands

**Keywords:** Learning and memory, Learning algorithms

## Abstract

Adolescence is characterized by a surge in maladaptive risk-taking behaviors, but whether and how this relates to developmental changes in experience-based learning is largely unknown. In this preregistered study, we addressed this issue using a novel task that allowed us to separate the learning-driven optimization of risky choice behavior over time from overall risk-taking tendencies. Adolescents (12–17 years old) learned to dissociate advantageous from disadvantageous risky choices less well than adults (20–35 years old), and this impairment was stronger in early than mid-late adolescents. Computational modeling revealed that adolescents’ suboptimal performance was largely due to an inefficiency in core learning and choice processes. Specifically, adolescents used a simpler, suboptimal, expectation-updating process and a more stochastic choice policy. In addition, the modeling results suggested that adolescents, but not adults, overvalued the highest rewards. Finally, an exploratory latent-mixture model analysis indicated that a substantial proportion of the participants in each age group did not engage in experience-based learning but used a gambler’s fallacy strategy, stressing the importance of analyzing individual differences. Our results help understand why adolescents tend to make more, and more persistent, maladaptive risky decisions than adults when the values of these decisions have to be learned from experience.

## Introduction

Although adolescence is a developmental period of good physical health and strength, it is associated with relatively high morbidity and mortality rates^1^. This downside of adolescence is largely due to an increased engagement in hazardous activities—such as dangerous behavior in traffic, substance and alcohol abuse, and unprotected sex^2–6^—which is often referred to as risk taking^7^. Indeed, risk taking has been defined in the developmental literature as the engagement in behaviors with potentially undesirable outcomes^8^. Importantly, however, some risky actions are also associated with beneficial outcomes that outweigh the potential negative ones, in which case risk taking can be advantageous. Participating in an international exchange program, for example, may result in failure and disappointment, but is more likely to promote personal growth and happiness; hence is a form of risk taking that is generally considered positive. Thus, to optimize positive and minimize negative life outcomes, adolescents’ ability to dissociate advantageous from disadvantageous risky behaviors may be more important than their inclination to take risk per se.

In many daily-life situations, one’s a priori knowledge about the potential outcomes of risky actions, and their probabilities, may be vague or incomplete. For example, although an adolescent may know that excessive alcohol consumption can have both positive and negative outcomes, he or she may not have a clear idea of the likelihood and severity of those outcomes. In situations like this, people can rely on experience-based learning to obtain a better estimate of risky actions’ expected value (i.e., the sum of all possible outcomes multiplied by their respective probabilities). This learning process allows people to optimize their risky choice behavior over time. For instance, by repeatedly experiencing that binge drinking results in bad hangovers, someone may learn that these negative outcomes outweigh the positive ones and therefore decide to stop binge drinking.

There is extensive evidence that experience-based learning processes differ between adolescents and adults^9–16^, and most of these studies suggest that adolescents are suboptimal learners^17^. Relating these findings to the risk-taking domain, it is an interesting possibility that developmental changes in learning may contribute to the increased incidence of maladaptive risk-taking behaviors in adolescents as compared to adults. Broadly consistent with this idea, two task features that have been found to promote risk taking in adolescents are also key ingredients of experience-based learning: Uncertainty about outcome probabilities (i.e., ambiguity)^18–21^ and the presence of outcome feedback following each choice^7,22,23^.

Many developmental studies have examined risk taking independent from learning using ‘decisions from description’ tasks that involve choices between two options—e.g., gambles or lotteries—that differ in their degree of risk^24–30^. Experience-based learning is not required in these tasks because participants are provided with complete information about all possible outcomes and their corresponding probabilities, creating a situation of explicit or known risk. Recent studies have also used tasks with incomplete information about the risky option’s outcome probabilities, creating a situation of unknown risk or ambiguity^18–21^. However, as these tasks did not include outcome feedback, ambiguity reduction through experience-based learning was not possible. Together, the results from these studies suggest that, in choice situations that do not involve learning, adolescents make similar choices as adults when choice options differ in known risk, but are more likely to choose ambiguous options.

In another class of risk-taking tasks—including the Balloon Analogue Risk Task (BART)^31^—participants decide when to stop a series of increasingly risky choices, in a stepwise manner, and receive positive or negative outcome feedback following each choice. Some studies have found that adolescents take more risk than adults on these tasks^22,23^. The optimal stopping point in the BART can in principle be learned from experience over the course of the task. However, with two recent exceptions^32,33^, developmental studies using this task focused on average measures of risk taking across all trials, leaving open whether and how developmental changes in experience-based learning may contribute to age-related differences in risk taking.

This question has been addressed, however, by studies using the Iowa Gambling task (IGT)^34^ or child-friendly versions of this task^35^, which measure risk taking in an experience-based learning context. Participants in the IGT make repeated choices between four options, represented as decks of cards. Two options are more risky (higher outcome variability) and have a low expected value, whereas the other two options are less risky (lower outcome variability) and have a high expected value. Participants do not receive prior information about the options’ outcome magnitudes or probabilities, but have to learn which options are most beneficial through trial-and-error. Healthy adults typically develop a preference for (one of) the advantageous, less risky, choice options over the course of the task, and developmental studies have found that adolescents’ choice behavior improves less over time^36–38^. These findings have been taken to suggest that adolescents, as compared to adults, are impaired at learning the negative value of risky choices, resulting in more persistent maladaptive risky choice behavior.

However, the IGT is a rather complex task, and people’s performance on this task reflects multiple cognitive processes that are difficult to disentangle^7,39^. First, as the risky options are also the disadvantageous ones (i.e., those with lower expected value), it is difficult to determine whether a more persistent preference for these options reflects a stronger tendency to make risky choices, a weaker sensitivity to expected value, and/or an impairment in experience-based learning. Second, the IGT requires participants to track and integrate gain and loss magnitudes and probabilities for four different choice options in parallel, which places a high demand on working memory. As working-memory function and its underlying neural circuitry continue to mature into late adolescence^40–44^, age-related differences in IGT performance may also reflect these developmental changes in working memory.

### Present study

In the present preregistered study, we aimed to examine potential differences between adolescents (12–17 years old) and young adults (20–35 years old; matched with regard to educational level) in the learning component of risk taking, while avoiding the interpretation difficulties associated with the IGT. To this end, we developed a simple task that requires people to learn the expected value of a risky choice option based on its previously experienced outcomes. Participants in this task make repeated choices between two options: a ‘sure’ (riskless) option and a risky option. While choices for the sure option always result in a fixed small reward, choices for the risky option can result in either a larger reward or no reward at all. Because participants are repeatedly presented with the same choice options, and directly observe the outcome of each choice, they can learn the expected value of the risky option over time and adjust their choice behavior accordingly. To minimize working-memory demands, participants are fully informed about the reward magnitudes for the sure and the risky option, and about the sure option’s reward probability (100%); hence the only variable that has to be learned is the risky option’s reward probability. Importantly, the risky option’s reward probability varies across task blocks, such that the expected value of the risky option is lower than, higher than, or identical to that of the sure option, in different blocks (referred to as risk-disadvantageous, risk-advantageous, and risk-neutral blocks, respectively). This aspect of the task allows us to dissociate effects of expected value and risk, and makes it more representative of real life in which risk taking is sometimes adaptive and sometimes maladaptive.

We expected participants to choose the risky option more often than the sure option during the first trials of each block, as this would allow them to estimate the risky option’s expected value. Successful learning should then cause a decrease in risky choice behavior over trials in risk-disadvantageous blocks, but not in risk-advantageous blocks (a trial x block type interaction). Regarding developmental differences, we expected that adolescents would be less efficient learners than adults^17^, and would therefore show a smaller adjustment of their risky choice behavior over trials as a function of the risky option’s expected value (a trial x block type x age group interaction). Given evidence that adolescents are more tolerant to ambiguity than adults^18,19,21^, we also expected adolescents to choose the risky option more often overall. Although we did not have specific hypotheses about the difference between early and mid-late adolescents in our task, work using related tasks has suggested differences between early and late adolescents^7^. To explore this issue, we administered our task to groups of early and mid-late adolescents.

We tested the hypotheses using multilevel regression. In addition to the regression analysis, we applied a set of computational models to participants’ choice data. Whereas the regression analysis tests for the presence of developmental differences in risk taking and learning efficiency, computational models of learning and decision-making can elucidate the nature of these differences. Specifically, we tested which out of several learning models—which differ in the complexity/optimality of the learning process and in how they can give rise to risk-sensitive behavior in our task—best explained each age group’s choice data. When possible, we also examined whether the estimated model parameters differed between the age groups.

## Results

### Preregistration

We preregistered our main research questions, analyses, and exclusion criteria using AsPredicted (http://aspredicted.org/kw96t.pdf). Non-preregistered analyses are treated as exploratory, and indicated as such.

### Experimental design

**Figure 1:**
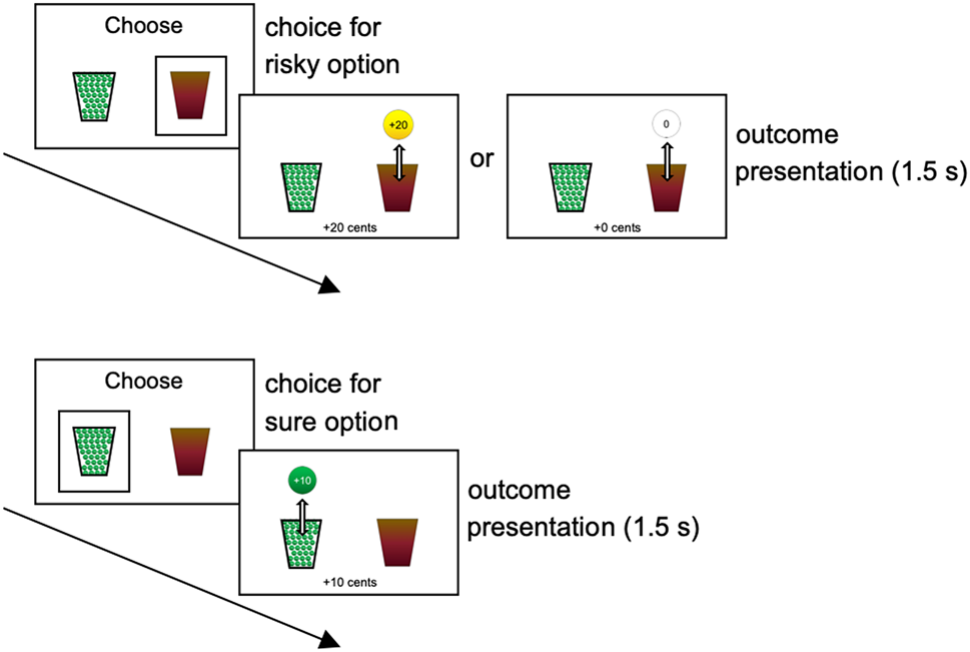
Trial outline in case of a risky (upper plot) and sure (lower plot) choice. This figure was created using Microsoft PowerPoint 16.16.27, https://www.microsoft.com/en-us/microsoft-365/powerpoint.

In each task block, participants made 20 choices between a sure and a risky option to earn monetary rewards. These options were depicted as vases filled with balls (Fig. 1; see Methods for task details). The ‘sure’ vase only contained balls with a ‘ + 10’ label (worth €0.10). The ‘risky’ vase contained a mix of balls with a ‘0’ label (worth €0) and balls with a ‘ + 20’ label (worth €0.20), with unknown proportions. Participants completed ten blocks of this task. The proportion of risky choices that yielded a 20-cents outcome was either 0.3 (four blocks), 0.7 (four blocks), or 0.5 (two blocks), such that the expected value of the risky option was, respectively, lower than (6 cents), higher than (14 cents), or identical to (10 cents) that of the sure option. Participants were not informed about the risky option’s expected value, but could acquire an estimate of this value over the course of each block based on the experienced outcomes.

To assess developmental trajectories of risk-taking behavior, we administered our task to groups of early adolescents (12–14-year-olds; N = 31), mid-late adolescents (15–17-year-olds; N = 39) and adults (20–35-year-olds; N = 35), and tested for linear and quadratic age effects across these three groups.

### Many participants showed evidence of a gambler’s fallacy-like strategy

We preregistered to exclude participants who switched to the sure option after experiencing a win outcome from the risky option on more than 30% of those trials, as this behavior is indicative of a gambler’s fallacy instead of a learning strategy. This exclusion criterion resulted in the exclusion of 46% of the adults, 67% of the mid-late adolescents and 68% of the early adolescents. Although the proportion of participants showing gambler’s fallacy-like behavior was numerically larger in the adolescent groups than in the adult group, a non-preregistered and thus exploratory analysis showed that this difference was not significant (${\upchi }^{2}$(2, n = 105) = 4.5, *p* = 0.11).

To verify that this behavior was maladaptive in the current task, we compared overall choice accuracy (proportion of choices for the option with the highest expected value, across all trials of the risk-advantageous and risk-disadvantageous blocks) for participants who showed vs. did not show this behavior. This non-preregistered and thus exploratory analysis showed that overall choice accuracy was lower in the participants who showed gambler’s fallacy-like behavior (as defined by our exclusion criterion) than in the participants who did not (49% vs. 62%, *t*(58.7) = 7.3, *p* < 0.001), confirming the maladaptive nature of this behavior.

Next, we report three sets of analyses: (i) the preregistered regression analysis examining effects of trial, block type and age group on risky choices; (ii) the preregistered computational-modeling analyses (Methods; Supplemental Text 1); and (iii) a non-preregistered hence exploratory latent mixture-model analysis examining individual differences in strategy use (Methods; Supplemental Text 2). In the first two analyses we excluded all participants who met our preregistered exclusion criterion. In the third analysis, we included all participants. In addition, we repeated our preregistered regression analysis on a larger number of participants based on the mixture-model results (Supplemental Text 3).

### The adaptive optimization of risky choice behavior improves from early adolescence to mid-late adolescence to adulthood

**Figure 2:**
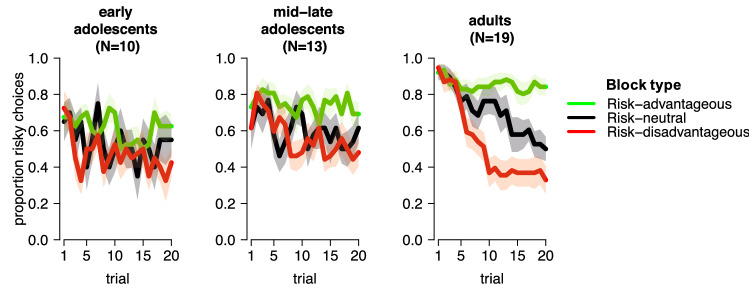
Mean proportion of choices for the risky option per trial, block type, and age group. Shaded areas represent 1 SEM. This figure was created using RStudio 1.1.463, https://www.rstudio.com.

Participants made more risky choices when the risky option had a higher expected value (main effect of block type, *z* = 19.2, *p* < 0.001; Fig. 2). This effect emerged over trials in a nonlinear way (block type x trial-linear interaction, *z* = 7.8, *p* < 0.001; block type x trial-quadratic interaction, *z* = 3.2, *p* = 0.002), suggesting that participants learned to optimize their risk choice behavior over time. Regarding developmental differences, the speed at which participants optimized their choice behavior over trials increased across the three age groups in a linear way, as reflected in block type x trial-linear x age group-linear (*z* = 6.1, *p* < 0.001) and block type x trial-quadratic x age group-linear (*z* = 3.6, *p* < 0.001) interactions. Thus, adults optimized their risky choice behavior faster than mid-late adolescents, who in turn optimized faster than early adolescents.

The overall number of risky choices increased linearly across the three age groups as well (main effect of age group-linear, *z* = 2.9, *p* = 0.004). Thus, contrary to our hypothesis, the number of risky choices was lowest in the early adolescents and highest in the adults. Figure 2 shows that this was driven by more risky choices in older age groups when the risky option was advantageous and, in all conditions, during early trials.

As adolescents optimized their choice behavior rather poorly over trials, we performed additional, exploratory, analyses to test some alternative explanations for poor performance. Specifically, we ruled out that adolescents carried over the learned risk value from one block to the next (Supplemental Fig. 6), and that they stopped engaging as the task progressed (Supplemental Text 6).

### Same analysis using a larger sample

To examine the robustness of the effects reported above, we repeated the analysis while including a larger number of participants—18 early adolescents, 25 mid-late adolescents and 28 adults—based on a less conservative exclusion criterion (see last Results section). Inclusion of this larger number of participants did not change the significance of the age-related effects reported above, with one exception: The overall number of risky choices no longer differed across the three age groups (main effect of age group-linear, *z* = 1.7, *p* = 0.09). However, the block type x trial x age group-linear interactions remained highly significant (*p*’s < 0.001). Results from this non-preregistered, hence exploratory, analysis are fully reported in Supplemental Text 3 and Supplemental Fig. 1.

### Computational modeling results

The regression results reported above suggest that the adaptation of risky choice behavior based on previously experienced outcomes improves between early and mid-late adolescence, and further improves between mid-late adolescence and adulthood. To examine whether these results reflect the use of different learning strategies, different parameter values of the same learning strategy, and/or differences in choice stochasticity across the different age groups, we applied a set of computational models to each age group’s choice data, using a hierarchical Bayesian approach. Full descriptions of the models and their equations can be found in the Methods and Supplemental Text 1; here we provide a brief overview.

We used two broad families of learning models: Reinforcement-learning models (Models 1A-C) and Bayesian ideal-observer models (Models 2A-D). Bayesian ideal-observer models take into account the uncertainty of expectations, and are more sophisticated and flexible but also more computationally demanding than reinforcement-learning models. Based on evidence that adolescents use suboptimal learning strategies^14,17^, we reasoned that adolescents’ choices may be better described by reinforcement-learning models.

Within each model family, we compared different model versions (Table 1). The first version of each model family (Models 1A and 2A) does not contain an explicit mechanism to explain risk-sensitive behaviors. The second version of each model family (Models 1B and 2B) can account for risk-sensitive behavior via asymmetric learning from win and no-win outcomes^9,45^. The third version of each model family (Models 1C and 2C) can account for risk-sensitive behavior via nonlinear subjective utilities for different outcome magnitudes^46^. Finally, we included a fourth version of the Bayesian ideal-observer model (Model 2D) which can account for risk-sensitive behavior via positive or negative effects of uncertainty on expected value^47,48^. This last version cannot be implemented in reinforcement-learning models, as these do not represent uncertainty.

**Table 1:** Overview of the learning models. The initial value of the risky option $\left( {{\text{Q}}_{1} } \right)$ was a free parameter in all models as well (not included in the table).

	Parameters	
	Reinforcement learning (1)	Bayesian ideal-observer (2)
Basic models (A)	Learning rate α	Update rate π
Asymmetric learning (B): Stronger weighting of win outcomes promotes risk seeking	Learning rates for win and no-win outcomes: α_+_ and α_−_	Update rates for win and no-win outcomes: π_+_ and π_−_
Nonlinear utility function (C): Overvaluation of higher outcomes promotes risk seeking	Utility parameter ${\upkappa }$ ${\upkappa }$ > 1 and ${\upkappa }$ < 1 cause over- and undervaluation of higher outcomes, respectively	Utility parameter ${\upkappa }$
Uncertainty affects value (D): Uncertainty bonus promotes risk seeking	Not applicable	Uncertainty parameter ${{\varphi }}$ ${{\varphi }}$ > 0 and ${{\varphi }}$ < 0 cause uncertainty bonus and penalty, respectively

We combined all learning models with a softmax decision function, which translates expected values into choice probabilities. The ‘inverse-temperature’ parameter controls the sensitivity of choice probabilities to differences in expected value. As the value of the inverse-temperature parameter increases, the probability that the option with the highest expected value is chosen also increases (i.e., choice stochasticity decreases).

To verify that our models were distinguishable and thus that potential developmental differences are meaningful^49^, we performed a model-recovery analysis. This analysis indicated that our procedures were able to distinguish between reinforcement-learning and Bayesian ideal-observer models and between different risk-sensitive mechanisms with rather high accuracy (Supplemental Text 4; Supplemental Fig. 5).

### Model comparison

**Figure 3:**
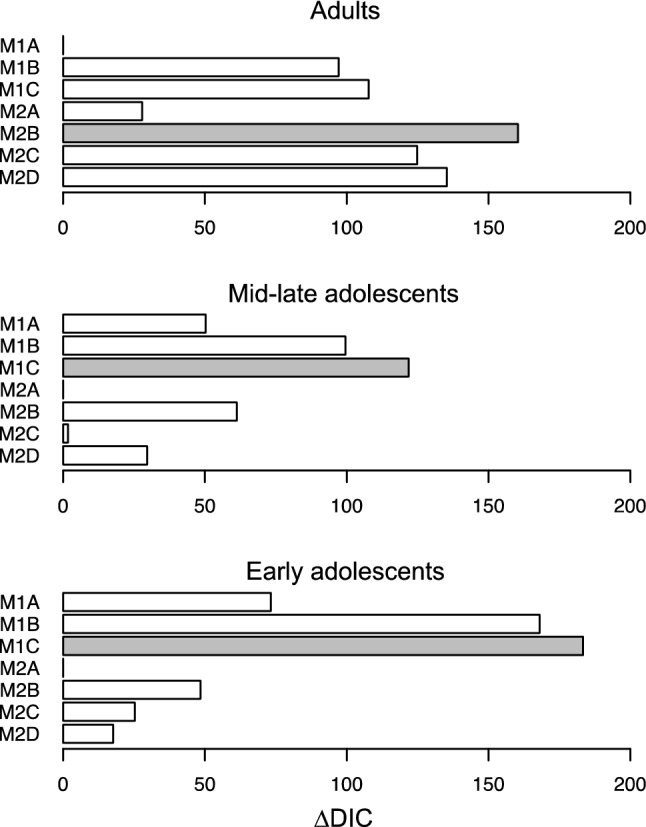
Model comparison. ∆DIC values reflect each model’s goodness of fit relative to the worst-fitting model. The winning model in each age group is indicated in gray. M1A = basic reinforcement-learning, M1B = reinforcement-learning with 2 learning rates, M1C = reinforcement-learning with nonlinear utility function; M2A = basic Bayesian ideal-observer, M2B = Bayesian ideal-observer with 2 update rates, M2C = Bayesian ideal-observer with nonlinear utility function, M2D = Bayesian ideal-observer with uncertainty bonus. This figure was created using RStudio 1.1.463, https://www.rstudio.com.

We computed ΔDIC values by subtracting each model’s DIC from the DIC of the worst-fitting model, such that higher ΔDIC values indicate a better fit (Fig. 3). For the adults, the Bayesian ideal-observer models outperformed their corresponding reinforcement-learning models. Conversely, for the early and mid-late adolescents’ data, the reinforcement-learning models outperformed their corresponding Bayesian ideal-observer models. This suggests that the adults reduced their learning rate over trials (within each block) as their expected-value estimates became more certain, whereas the adolescents used a constant learning rate. Note that reducing one’s learning rate over trials is optimal when stimulus-outcome contingencies are stable, as in the current task^14^. Thus, the adolescents used a simpler, suboptimal, learning process than the adults. Furthermore, models that included a mechanism to account for risk-sensitive behavior (models 1B-C and 2B-D) performed better than their corresponding basic models (models 1A and 2A), in all age groups. The winning model for the adults was the Bayesian ideal-observer model with two update rates (Model 2B), and the winning model for both adolescent groups was the reinforcement-learning model with nonlinear utility function (Model 1C).

Supplemental Fig. 2 illustrates the fit of the winning models for each age group. We inspected the differences in model fit between the two model families visually and also compared the absolute deviation between the simulated and observed proportion of risky choices (Supplemental Fig. 2). Both inspections did not provide clear clues as to why the reinforcement-learning model with nonlinear utility function fitted the data better in both adolescent groups and the Bayesian ideal-observer model with asymmetric updating fitted better in the adults. We speculate that the difference in DIC values is due to individual differences in strategy use, as supported by our latent-mixture model analysis (see below).

### Parameter estimates

**Figure 4:**
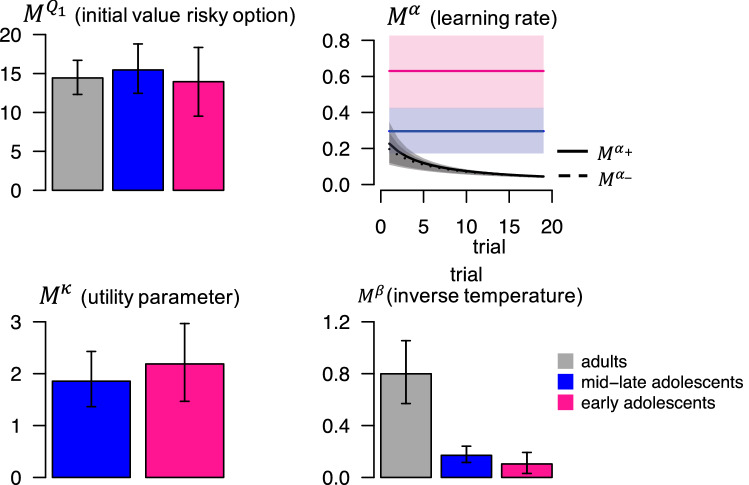
Posterior medians of the winning models’ group-level mean parameters, per age group. Error bars and shaded areas indicate 95% HDIs of the posterior distributions. This figure was created using RStudio 1.1.463, https://www.rstudio.com. Note ${\text{ Q}}_{1} { }$ is expressed in cents. For the adult group, ${\text{Q}}_{1}$ was computed using the posterior median of the initial a parameter $\left( {{\text{a}}_{1} } \right)$ of the winning Bayesian ideal-observer model, and trial-specific learning rates α_+_ and α_−_ were computed using the posterior medians of the update-rate parameters (π_+_ and π_−_) of the winning Bayesian ideal-observer model (Supplemental Text 1). The lines for α_+_ and α_−_ almost overlap.

To shed more light on the learning and choice mechanisms implemented in the winning model for each age group, we next examined the group-level mean (M) parameters of these models. Figure 4 shows the medians and 95% highest density intervals (HDIs) of these parameters’ posterior distributions. Supplemental Fig. 3 shows the full posterior distributions.

The posterior distribution of the risky option’s initial expected value $\left( {{\text{M}}^{{{\text{Q}}_{1} }} } \right)$ was higher than 10—the value of the sure option—in all age groups. Such ‘optimistic initial values’ for the risky option promote exploration of this option, in line with participants’ initial preference for the risky option (Fig. 2).

The posterior distribution of the learning-rate parameter $\left( {{\text{M}}^{{\upalpha }} } \right)$ was higher for the early adolescents than for the mid-late adolescents (99.6% of the difference distribution lay above 0), suggesting that the early adolescents’ value estimate for the risky option was driven more by the most recent outcome. The adults’ data was best explained by a dynamic learning rate, which started out at a similar value as the mid-late adolescents’ learning rate and then decreased over trials. Although the best-fitting model for the adults contained separate update rates for positive and negative outcomes, the group-level means of these parameters were highly similar (Fig. 4, solid vs. dotted black line in upper right plot). This suggests that some adults learned more from positive than negative outcomes, while others showed the opposite bias, but that there was no systematic learning asymmetry (at the individual level, π_+_ was higher than π_−_ for 9 adults, and π_+_ was lower than π_−_ for 10 adults).

The adolescents’ data was best explained by a model with nonlinear utility function, and the posterior distribution of the parameter governing the shape of the utility function $\left( {{\text{M}}^{{\upkappa }} } \right)$ lay above 1 for both adolescent groups. This suggests that adolescents’ subjective value of a 20-cent outcome was more than twice the subjective value of a 10-cent outcome (convex utility function). This overvaluation of the higher outcomes promotes risk taking, which in our task should impair performance in the risk-disadvantageous blocks but improve performance in the risk-advantageous blocks. However, an advantage for the adolescents over the adults in the risk-advantageous blocks was not apparent in the choice data (Fig. 2), likely due to adolescents’ higher choice stochasticity, as described below.

The posterior distribution of the inverse-temperature parameter $\left( {{\text{M}}^{{\upbeta }} } \right)$ was higher in the adults than in both adolescent groups, but did not differ between the early and mid-late adolescents (9% of the difference distribution lay above 0). The lower inverse temperature in the adolescents than the adults may reflect that that adolescents’ choices were driven less by the options’ expected-value estimates (i.e., they were less prone to choose the option with the highest expected-value estimate), reflecting more stochastic choice behavior. Alternatively, the lower inverse temperature in the adolescents may reflect that the value-updating algorithms implemented in our models were less able to capture the adolescents’ choices. Adolescents’ lower inverse temperature can explain why they made fewer risky choices during the initial trials of a block: Even though the risky option’s initial expected value was similarly optimistic in all age groups, adolescents’ choices were less sensitive to expected values, which resulted in a weaker initial choice preference for the risky option. Adolescents’ low inverse temperature may also explain why their overvaluation of the higher win outcomes (convex utility function)—which should increase the expected value of the risky option—was not translated in a substantial increase in risky choices.

In sum, our modeling results identified two mechanisms underlying the impaired adaptation of risky choice behavior over time in adolescents as compared to adults: (i) adolescents used a simpler, suboptimal, learning process involving a constant learning rate; and (ii) adolescents showed a higher degree of choice stochasticity (lower inverse temperature). In addition, the results suggest that the adolescents, but not the adults, overvalued the highest outcomes. The impaired performance of the early compared to the mid-late adolescents may be due to the elevated learning rate (causing excessive expectation updating) and numerically lower inverse temperature in the early adolescents.

### Results of latent-mixture model analysis including all participants

We performed an additional (not preregistered hence exploratory) latent mixture-model analysis to infer the learning or non-learning strategy used by each individual participant, and to examine whether the prevalence of specific strategies differed between our three age groups (Supplemental Text 2). In this analysis, we included all participants.

Our mixture model included the two winning learning models from the previous analysis (reinforcement-learning model with nonlinear utility function and Bayesian ideal-observer model with two update rates). In addition, it included two gambler’s fallacy models. These were identical to the two learning models except that they used negative learning/update rates. Thus, the gambler’s fallacy models assume that the value of the risky option decreases following each win outcome and increases following each no-win outcome, reflecting the belief that outcomes that occurred more frequent in the past will be less likely in the future. Finally, we included a fifth model that does not consider the experienced outcomes. This model—the epsilon-risky model—has a fixed probability of choosing the risky option on each trial (determined by parameter ε), hence can account for guessing behavior and general tendencies to seek or avoid risk that are insensitive to outcome feedback.

**Figure 5:**
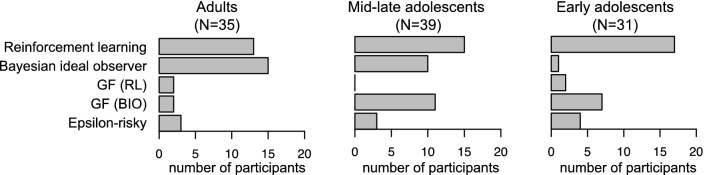
Inferred strategy use according to our mixture-model analysis. The plots show the number of participants per age group assigned to each of the 5 models included in the mixture model. GF = gambler’s fallacy, RL = reinforcement learning, BIO = Bayesian ideal observer. This figure was created using RStudio 1.1.463, https://www.rstudio.com.

Figure 5 shows the number of participants in each age group assigned to each model (Supplemental Fig. 4 shows the model assignment per participant). The proportion of participants assigned to a learning model (reinforcement learning or Bayesian ideal-observer) did not differ across the three age groups (${\upchi }^{2}$(2, n = 105) = 4.0, *p* = 0.14).

For the participants assigned to a learning model, the proportion of participants assigned to the reinforcement-learning vs. Bayesian ideal-observer model differed across the three age groups (${\upchi }^{2}$(2, n = 71) = 11.1, *p* = 0.004). While the proportion of participants assigned to the Bayesian ideal-observer model was highest in the adults and lowest in the early adolescents, the proportion of participants assigned to the reinforcement-learning model showed the opposite pattern, corroborating the model-comparison results from the previous analysis.

Finally, a few participants in each age group were assigned to the epsilon-risky model, suggesting that their tendency to choose the risky option was constant over time and independent of the experienced outcomes.

#### Comparison of mixture-model results and preregistered exclusion criterion

The number of participants assigned to a learning model by our mixture-model analysis (58% of the early adolescents, 64% of the mid-late adolescents, and 80% of the adults) was higher than the number of participants who used a learning strategy according to our preregistered criterion (32% of the early adolescents, 33% of the mid-late adolescents, and 54% of the adults). Our preregistered criterion assumed that participants who frequently switched to the sure option after a win outcome from the risky option did not use a learning strategy. However, frequent win-switch behavior does not necessarily reflect a lack of learning, but could also be due to a high degree of choice stochasticity, which may explain the discrepancy between our preregistered criterion and mixture-model results. As our computational models were designed to tease apart learning and choice processes, these are arguably better-suited to dissociate learners from non-learners than our rather crude preregistered criterion. Therefore, we repeated the regression analysis on risky choice behavior while including all participants who learned according to our mixture-model analysis (see ‘Same analysis using a larger sample’ section above, and Supplemental Text 3).

## Discussion

Adolescence is characterized by an increase in maladaptive risk-taking behaviors, but whether and how this relates to developmental changes in experience-based learning is largely unknown. We addressed this question using a simple learning task involving repeated choices between a sure and a risky option. Importantly, the expected value of the risky option—which could be learned from experience—varied across task blocks, allowing us to dissociate the learning-driven optimization of risky choice behavior from general risk-taking tendencies.

Our exploratory mixture-model analysis suggested that approximately two-thirds of the participants used a learning strategy, while the remaining participants used a gambler’s fallacy or other irrelevant choice strategy. Thus, a substantial proportion of the participants in each age group did not engage in the experience-based, incremental, type of learning that our task was designed to measure. This highlights the importance of considering individual differences in strategy use, for example using mixture models, before averaging data across participants. It also raises the questions whether and how the use of suboptimal (non-learning) strategies can be prevented, and to what degree such strategies contribute to maladaptive risk-taking in other learning tasks (such as the IGT) and in real life.

After excluding participants who used a gambler’s fallacy-like strategy (see Limitations and future directions section below), we found that adolescents adapted their risky choice behavior less to the expected value of this behavior than adults. Computational modeling provided evidence that both adolescent groups, compared to the adult group, used (i) a simpler, suboptimal, learning process (involving a constant learning rate) and (ii) a more stochastic choice policy. The modeling results also suggested that adolescents, but not adults, overvalued the highest rewards. However, this last effect was overshadowed by adolescents’ larger choice stochasticity such that a corresponding increase in risk taking was not observed. Together, these findings suggest that adolescents’ suboptimal risky choice behavior was largely due to an inefficiency in core learning and choice processes, and did not reflect increased risk seeking per se.

We found that the optimization of risky choice behavior improves during adolescence as well. Early adolescents performed worse than mid-late adolescents, which could be explained by early adolescents’ higher constant learning rate (causing excessive expectation updating) and more stochastic choice policy. Our findings thus suggest that the “settings” of learning and choice parameters improve between early and mid-late adolescence, and that further optimization—the transition to a Bayesian learning process (involving a dynamic learning rate) and a further decrease in choice stochasticity—occurs between mid-late adolescence and adulthood.

Our results are consistent with findings from the IGT that adolescents develop a weaker preference for the advantageous choice options than adults^36–38^, and suggest that adolescents’ suboptimal IGT performance may be related to (i) suboptimal learning strategies, (ii) enhanced choice stochasticity, and/or (iii) overvaluation of higher rewards. Our results are also consistent with two previous studies that examined the role of learning in adolescent risk taking on the BART^32,33^. Adolescents in these studies showed a weaker optimization of their risk-taking behavior (number of pumps) over the course of the task than adults, in line with our findings, suggesting that this developmental effect generalizes across different risk-taking paradigms.

There is an interesting discrepancy between our results and those from previous developmental studies examining ‘decisions from description’^18,19,21^. These previous studies, which did not involve learning, found that adolescents were more willing to choose options with unknown outcome probabilities than adults, which was attributed to adolescents’ larger tolerance for ambiguity. In contrast, during the first trial of each block in our study (i.e., before learning had occurred), adolescents chose the option with unknown outcome probabilities less often than adults. These opposite developmental effects suggest that age-related differences in ambiguity attitude are not fixed across situations, but depend on whether or not ambiguity can be reduced through learning (also see^50^). In our task, choosing the risky option during the first trials allowed participants to quickly estimate its expected value and thereby optimize future choices. That adolescents did this less often than adults is thus indicative of suboptimal, less goal-directed, (learning) behavior. Our modeling results suggested that this behavior was not due to a lower initial valuation of the risky option, but reflected adolescents’ more stochastic choice policy which made them less prone to choose the option with the highest expected value.

A higher degree of choice stochasticity in younger age groups—as reflected in a lower inverse-temperature parameter—is a common finding in experience-based learning tasks^10,13,14,38,51,52^. This has been attributed to children’s and adolescents’ increased tendency to explore lower-valued but more uncertain options^15^. However, in our task, the adolescents chose the risky (and more uncertain) option less often than the adults, which is inconsistent with the notion of increased exploration. Instead, adolescents’ more stochastic choice behavior in our task may simply reflect a higher degree of choice randomness, possibly related to attentional or motivational lapses. Alternatively, adolescents’ more stochastic choice behavior may also reflect an increased tendency to alternate between the choice options, regardless of how rewarding they are. Finally, it could be that adolescents more often held specific, idiosyncratic, beliefs about the nature of the task (e.g., the belief that a series of two good outcomes will be followed by a bad outcome), preventing them from learning the true values of the risky option and leading to an apparently high degree of choice stochasticity.

### Limitations and future directions

An important open question concerns the degree to which the developmental effects found in our task generalize to real-life situations. Recent evidence suggests that developmental changes in learning are most pronounced in more demanding tasks, such as those with a higher cognitive load^53,54^. This suggests that the age-related differences in learning and choice mechanisms revealed in our relatively simple task may have even stronger effects on risk taking in more complex environments, such as those encountered in real life. However, most real-life risk-taking scenarios also involve social and emotional factors that were not captured by our task. For example, adolescents (but not adults) have been shown to take more risk when observed by peers, which has been attributed to a heightened sensitivity to the potential rewards of risky actions^55–57^. Thus, peer pressure likely increases risk seeking in adolescents via reward-valuation mechanisms not identified in our study. Whether peer pressure also affects adolescents’ learning process, utility function, and/or choice stochasticity—and thereby the ability to optimize risky decisions over time—is an interesting question for future studies.

Another limitation of our task is that the worst possible outcome was gaining nothing (0 cents), but participants could never lose money. Like several previous studies^18,19,24,45^, we thus focused on risk taking in the gain domain. As people are generally risk-averse in the gain domain but risk-seeking in the loss domain^58,59^, we expect that participants in a loss version of our task would make more risky choices overall. Whether outcome valence also affects people’s learning and choice processes, and whether outcome-valence effects change during development, is currently unknown. To address these questions, future developmental studies could compare gain and loss versions of our task.

Finally, due to the substantial proportion of participants engaging in irrelevant choice strategies, the sample size of our computational analyses was rather small, potentially affecting the reliability of our results^60^. However, one of the advantages of our hierarchical modeling approach, and especially our Bayesian framework^61,62^, is that valid results can even be obtained with small sample sizes^63^. This theoretical account in combination with rather high model recoverability (Supplemental Text 4) strengthen the reliability of our results, regardless of the small sample size. Nevertheless, future studies are advised to replicate our results using larger sample sizes, especially when aimed at further investigation of individual differences (e.g.,^64^).

To conclude, by combining a novel experience-based risk-taking task with computational modeling, we demonstrated how adolescents’ simpler learning process and more stochastic choice policy can give rise to an impaired optimization of risky choice behavior over time. Future work could examine the generalizability of our findings to risk-taking behaviors in social contexts and in the loss domain, which may eventually inform interventions aimed at reducing the detrimental outcomes associated with adolescents’ maladaptive forms of real-life risk taking.

## Methods

### Participants

A total of 35 adults (mean age = 22.6; age range = 20–35; 63% female), 39 mid-late adolescents (mean age = 15.8; age range = 15–17; 74% female), and 31 early adolescents (mean age = 13.0; age range = 12–14; 48% female) completed the study. The adults were students, or former students, at universities or colleges of higher professional education. The mid-late adolescents were in the fourth or fifth year of high school, and the early adolescents were in the first or second year of high school (all pre-university or higher general secondary education).

Participants reported no history of psychiatric or neurological disorders. Adult participants received a fixed amount of €2.50 or course credits for their participation, plus a variable performance-dependent amount of maximally €2.50. Adolescent participants only received the variable performance-dependent amount of maximally €2.50 (the school we collaborated with did not allow larger rewards). All participants of age 16 and older provided written informed consent. Primary caretakers of participants younger than 16 were informed about the experiment and provided active written informed consent. All procedures were approved by the ethics committee of the Faculty of Social and Behavioural Sciences of the University of Amsterdam, and the study was performed in accordance with the relevant guidelines and regulations.

We preregistered to exclude participants who switched to the sure option after experiencing a win outcome from the risky option on more than 30% of those trials, as we reasoned that this behavior is indicative of a gambler’s fallacy instead of a learning strategy, reflecting a misunderstanding of the task. Based on this criterion, we excluded 16 adults (46%), 26 mid-late adolescents (67%), and 21 early adolescents (68%), leaving a final sample of 19 adults, 13 mid-late adolescents, and 10 early adolescents. We performed our preregistered analyses on this sample. In addition, we performed a non-preregistered, hence exploratory, latent mixture-model analysis (described in Supplemental Text 2) including all participants. This analysis suggested that 28 adults, 25 mid-late adolescents, and 18 early adolescents used a learning, instead of a gamblers fallacy, strategy—a larger number than suggested by our preregistered criterion. Therefore, we also repeated our behavioral analysis on this larger sample.

### General procedure

Adolescent participants were tested in a classroom or computer room at their high school, in groups of approximately 20 participants. Adult participants were tested in a room at the university, in groups of at least two participants. At least one experimenter was always present in the testing room as well. Participants performed the task individually on a laptop or PC. Before starting the task, participants received computerized task instructions and performed a short practice block. The task lasted approximately 15 to 20 min, after which participants were reimbursed.

### Experience-based risk-taking task

In each task block, participants made 20 choices between two options to earn monetary rewards. Choices for one option always paid off 10 cents; we refer to this option as the sure option. Choices for the other option paid off either 20 or 0 cents, we refer to this option as the risky option.

The two options were depicted as vases, displayed at the left and right side of the screen (Fig. 1). The ‘sure’ vase was filled with green balls with a ‘ + 10’ label (worth €0.10). This vase was transparent such that participants could see that it contained green balls only. The ‘risky’ vase was opaque such that its content was invisible, and participants were instructed that it contained a mix of white balls with a ‘0’ label (worth €0) and gold balls with a ‘ + 20’ label (worth €0.20). However, the proportion of white and gold balls in this vase was unknown. We instructed participants that each time they chose a vase, one ball would be drawn from that vase and displayed on the screen, and participants would gain the money associated with that ball. Participants were also instructed that after a ball was drawn, it was returned into its vase and all balls were shuffled for the next trial (i.e., random draws with replacement).

On each trial, participants chose a vase by pressing a left (‘z’) or a right (‘/’) key. A black frame appeared around the chosen vase and 200 ms later one ball was displayed above that vase. The ball moved upwards and then back downwards (back inside the vase) as if it was drawn from the vase and then returned. The ball-drawing animation lasted 1.5 s; during this period the payoff amount was also displayed at the bottom of the screen (+ 10 cents, + 20 cents, or + 0 cents).

Unbeknownst to the participants, the proportion of risky choices that yielded 20 cents was either 0.3 (four blocks), 0.7 (four blocks), or 0.5 (two blocks). Specifically, out of every ten risky choices, either three, seven, or five choices paid off 20 cents and the remaining choices paid off 0 cents, in random order. Thus, the expected value of the risky option was lower than (6 cents), higher than (14 cents), or identical to (10 cents) the expected value of the sure option (10 cents), respectively. We refer to these three block types as risk disadvantageous, risk advantageous, and risk neutral. Participants performed the ten blocks in random order, with one constraint: Both the first five and the last five blocks had to contain one risk-neutral, two risk-disadvantageous, and two risk-advantageous blocks. A new risky vase, with a unique color, was introduced in each block. We instructed participants that the number of gold and white balls inside the opaque vase would vary across blocks, but would not change during a block.

Except for the outcome probabilities of the risky option, participants were fully informed about the task structure and procedure. After completing the task, participants received the average amount of money they had gained across the ten blocks (i.e., total amount divided by ten, rounded to the nearest 50 cents). In addition, the adults also received their fixed payment of €2.50 or course credits.

### Behavioral analysis

We performed a multilevel logistic regression analysis on the single-trial choice data using the lme4 package^65^ in R. The dependent variable was the binary choice variable (risky vs. sure choices, coded as 1 vs. 0, respectively). We tested for effects of block type (coded as -1, 0 and 1 for the risk-disadvantageous, risk-neutral and risk-advantageous blocks), block-specific trial (linear and quadratic effects), age group (linear and quadratic effects), and their interactions. In addition to these fixed effects, we modeled random intercepts and random slopes for the within-subject fixed effects (separately for each age group). This model failed to converge. Therefore, as preregistered, we removed the random slopes and only modeled random intercepts for the within-subject fixed effects.

### Computational models

We examined the ability of a range of learning models to capture each age group’s trial-to-trial choice data. Supplemental Text 1 contains a detailed description of all models, the model equations, and our hierarchical Bayesian parameter estimation methods; here we provide an intuitive description of the models (also see Table 1).

All models estimate the expected value of the risky option—and update this estimate following each new outcome. Note that the expected value of the sure option did not have to be estimated, as its payoff was fixed and known in advance (100% probability of €0.10; hence its expected value is always 10 cents).

We used two broad families of learning models: Reinforcement-learning models (Q-learning models; Models 1A-C) and Bayesian ideal-observer models (beta-binomial models; Models 2A-D). Reinforcement-learning models represent expected values as point values, which are updated in response to prediction errors using a constant learning rate^66^. In contrast, Bayesian ideal-observer models represent expected win probabilities as beta distributions, naturally track both the mean and uncertainty of expectations^67–71^. These latter models determine their effective learning rate on each trial as a function of the current uncertainty (higher uncertainty results in stronger updating). Because they take into account the uncertainty of expectations, Bayesian ideal-observer models are more sophisticated and flexible but also more computationally demanding than reinforcement-learning models. Bayesian ideal-observer models have been shown to describe adults’ choices in a reward-learning task better than reinforcement-learning models^68^, but these two model families have not been compared in adolescent samples. Based on evidence that adolescents, as compared to adults, use suboptimal learning strategies^14,17^, we reasoned that adolescents’ choice data may be better described by reinforcement-learning models than Bayesian ideal-observer models.

Within each model family, we compared different model versions that vary in whether and how they can account for risk-sensitive behavior (Table 1). The first, most basic, version of each model family (Models 1A and 2A) does not contain an explicit mechanism to explain risk-avoidance or risk-seeking behaviors. These models have two free parameters: The learning or update rate which controls the speed of learning, and the initial expected value of the risky option which captures the initial preference for the risky option (before any learning has taken place). The other versions are extended versions of these basic models.

The second version of each model family (Models 1B and 2B) can account for risk-sensitive behavior via asymmetric learning. Specifically, it contains separate learning/update rates for win and no-win outcomes^9,45^. If win outcomes are weighted stronger than no-win outcomes, this leads to an overestimation of the risky option’s expected value, promoting risk seeking. The opposite learning asymmetry has the opposite effect, promoting risk avoidance.

The third version of each model family (Models 1C and 2C) does not allow for asymmetric learning, but allows for nonlinear subjective utilities for different win magnitudes^46^. The subjective utility of the risky option’s win outcome (20 cents) can be higher or lower than 20 cents (convex vs. concave subjective utility curve, respectively), depending on the setting of a utility parameter^45^. Values of this parameter smaller than 1 produce undervaluation of the risky option’s win outcomes, promoting risk aversion, whereas values of this parameter larger than 1 have the opposite effect.

Finally, we included a fourth version of the Bayesian ideal-observer model (Model 2D) which allows the value of the risky option to increase or decrease as a function of its uncertainty^47,48^. The expected value of the risky option is uncertain, especially during the first trials of each block, while the expected value of the sure option is not. Therefore, a positive effect of uncertainty (uncertainty bonus) will increase the value of the risky option and hence promote risky choices, whereas a negative effect of uncertainty (uncertainty penalty) will have the opposite effect. The direction and magnitude of the uncertainty effect is controlled by a free parameter. Note that an uncertainty bonus/penalty cannot be implemented in reinforcement-learning models, as these do not represent uncertainty.

### Decision function

We combined all learning models with a softmax decision function, which translates the expected-value difference of the risky and the sure option into a probability of choosing the risky option. The sensitivity of choice probabilities to differences in expected value is controlled by an ‘inverse-temperature’ parameter. If this parameter is 0, both options are equally likely to be chosen, irrespective of their expected values. As the value of the inverse-temperature parameter increases, the probability that the option with the highest expected value is chosen also increases.

### Model comparison

We compared the performance of the different models using the deviance information criterion (DIC)^72^, separately for each age group. The DIC is a hierarchical modeling generalization of the AIC which is easily computed in hierarchical Bayesian model-selection problems using Markov chain Monte Carlo (MCMC) sampling. It provides an index of the goodness of fit of a model, penalized by its effective number of parameters. Models with smaller DIC are better supported by the data.

### Exploratory latent-mixture model analysis

We performed an additional model-based analysis on all participants—including those who were excluded from the previous analyses—to examine the choice strategies used by each individual participant: A latent mixture-model analysis^73–75^. This non-preregistered hence exploratory analysis is described in Supplemental Text 2.

## Supplementary Information

Supplementary Information.
