# Supplementary material for: Impaired learning to dissociate advantageous and disadvantageous risky choices in adolescents
Source: Sci Rep. 2022 Apr 20;12:6490. doi: 10.1038/s41598-022-10100-7 (PMC9021244; doi:10.1038/s41598-022-10100-7)
Supplement: Supplementary file 1 — Supplementary Information. [file 41598_2022_10100_MOESM1_ESM.pdf]

Supplemental Text 1: Description of models and parameter-estimation methods

Model 1A: Basic reinforcement-learning model

On each trial t that the risky option is chosen, this model computes the prediction error δ_t, which is the difference between the reward attained, R_t, and the estimated expected value of the risky option at the onset of that trial, Q_t:

$$\delta_t = R_t - Q_t \quad [1.1]$$

This prediction error is used to update the estimated expected value of the risky option according to a standard reinforcement-learning algorithm [1]:

$$Q_{t+1} = Q_t + \alpha\delta_t \quad [1.2]$$

Learning-rate parameter α determines the impact of each new prediction error on the subsequent expectation.

The initial expected-value estimate of the risky option (on trial 1 of each block), Q_1, was estimated as a free parameter in all reinforcement-learning models (Model 1A-C) to capture participants' initial preference for the risky option.

Model 1B: reinforcement-learning model with two learning rates

This model differs from Model 1A in that it uses two separate learning rates (α_+ and α_-) to update expectations following positive and negative prediction errors [2,3]; hence Equation 1.2 is replaced by:

$$Q_{t+1} = \begin{cases} Q_t + \alpha_+\delta_t \text{ if } \delta_t > 0 \\ Q_t + \alpha_-\delta_t \text{ if } \delta_t < 0 \end{cases} \quad [2.1]$$

If $\alpha_+ > \alpha_-$, positive prediction errors (evoked by €0.20 outcomes from risky choices) result in stronger updating of the risky option's expected value estimate than negative prediction errors (evoked by €0 outcomes from risky choices). This will lead to an overestimation of the risky option's expected value, promoting risk seeking. The opposite learning asymmetry will have the opposite effect, promoting risk avoidance.

Model 1C: reinforcement-learning model with nonlinear utility function

This model differs from Model 1A in that it incorporates nonlinear subjective utilities, U, for different reward magnitudes [4]. Our task included three different reward magnitudes: 0, 10 and 20 cents. To allow for a nonlinear subjective utility curve, we assumed that U(0) = 0 ,

U(10) = 10, and U(20) = κ*20 [3]. Values of κ (the utility parameter) smaller than 1 are thus consistent with a concave subjective utility curve (undervaluation of the highest outcome that can be obtained from the risky option, promoting risk aversion), and values of κ larger than 1 are consistent with a convex utility curve (overvaluation of the highest outcome that can be obtained from the risky option, promoting risk seeking). In this model, the prediction error depends on the subjective utility of the reward rather than the actual reward, hence equation 1.1 is replaced by:

$$\delta_t = U_t - Q_t \qquad [3.1]$$

The update rule is the same as in the first model (equation 1.2).

Model 2A: Basic Bayesian ideal-observer model

According to this model, participants assume that the sequence of €0.20 (gain) and €0 (no gain) payoffs obtained from the risky option is the result of a Bernoulli process with a constant probability of gaining €0.20. This probability is represented as a beta distribution which is updated following each new observed outcome. Parameters a and b of the beta distribution represent evidence for, respectively, gaining and not gaining €0.20. These two possible outcomes, O, are coded as 1 and 0, respectively. Thus, following each risky choice, the model updates (increases) a after a win outcome, and b after a no-win outcome, with the update rate determined by parameter π:

$$a_{t+1} = a_t + \pi * O_t \qquad [4.1]$$

$$b_{t+1} = b_t + \pi * (1 - O_t) \qquad [4.2]$$

This results in a beta distribution that reflects the expected probability of gaining €0.20; it is biased towards 1 and 0 if, respectively, €0.20 and €0 outcomes occur most frequently, and becomes more precise as more outcomes are observed (i.e., as a + b increases). As the beta distribution becomes more precise (i.e., higher certainty), it will be affected less by each new outcome (i.e., a lower effective learning rate).

We assume that participants' expected probability of gaining 20 cents reflects the mean of the beta distribution on the current trial, μ_t:

$$\mu_t = \frac{a_t}{a_t + b_t} \qquad [4.3]$$

Finally, this probability is multiplied by 20 (the amount that can be gained from the risky option) to obtain the expected value estimate of the risky option (in cents):

$$Q_t = \mu_t * 20 \quad [4.4]$$

The initial values of a and b (a_1 and b_1), which determine the risky option's expected value estimate on trial 1 of each block, were estimated as free parameters in all Bayesian ideal-observer models (Models 2A-D), to capture participants' initial preference for the risky option. To prevent a tradeoff between the update-rate and initial-value parameters we constrained a_1 and b_1 to sum to 2. Thus, values of a_1 below and above 1 reflect that the initial expected probability of gaining €0.20 is lower and higher than 0.5, respectively.

Model 2B: Bayesian ideal-observer model with two update rates

Like Model 1B, this model uses two separate update rates (π_+ and π_-) to update expectations following win and no-win outcomes, hence Equations 4.1 and 4.2 are replaced by:

$$a_{t+1} = a_t + \pi_+ * O_t \quad [5.1]$$

$$b_{t+1} = b_t + \pi_- * (1 - O_t) \quad [5.2]$$

Thus, as in Model 1B, asymmetric learning from win and no-win outcomes can account for risk-sensitive behavior.

Model 2C: Bayesian ideal-observer model with nonlinear utility function

Like Model 1C, this model allows for a nonlinear subjective utility curve by assuming that U(0) = 0, U(10) = 10, and U(20) = κ*20. As in model 1C, values of κ smaller than 1 promote risk aversion, and values of κ larger than 1 promote risk seeking. This model differs from Model 2A in that Equation 4.4 is replaced by:

$$Q_t = \mu_t * U(20) \quad [6.1]$$

Model 2D: Bayesian ideal-observer model with uncertainty bonus/penalty

This model allows the value of the risky option to increase or decrease as a function of its uncertainty [5,6]. Specifically, it assumes that the uncertainty about the risky option's win probability is reflected in the variance of the current beta distribution, σ_t:

$$\sigma_t = \frac{a_t * b_t}{(a_t + b_t)^2 * (a_t + b_t + 1)} \quad [7.1]$$

According to this model, the expected value estimate of the risky option not only depends on the mean of the beta distribution, but also on its variance; hence equation 4.4 is replaced by:

$$Q_t = \mu_t * 20 + \varphi\sigma_t \qquad [7.2]$$

Parameter φ controls how uncertainty affects the expected value estimate. Values of $\varphi > 0$ correspond to an 'uncertainty bonus'. As the expected value of the risky option is uncertain while the expected value of the sure option is not, this will promote risky choices. In contrast, values of $\varphi < 0$ correspond to an 'uncertainty penalty', discouraging risky choices. Either effect will decrease as the risky option is selected more often and uncertainty (σ) decreases. Note that an uncertainty bonus/penalty cannot be implemented in a reinforcement-learning model, as these models do not represent uncertainty.

Softmax function

We combined all learning models with a softmax function, which computes the probability of choosing the risky stimulus on trial t, $P_{risky,t}$, as:

$$P_{risky,t} = \frac{e^{\beta Q_{risky,t}}}{e^{\beta Q_{risky,t}} + e^{\beta Q_{sure}}} = \frac{1}{1+e^{-\beta(Q_{risky,t} - Q_{sure})}} \qquad [8.1]$$

$Q_{risky,t}$ refers to the trial-specific expected value of the risky option (derived from the learning models described above), and Q_{sure} refers to the expected value of the sure option (fixed to 10 cents). Inverse-temperature parameter β controls the sensitivity of choice probabilities to the difference in expected-value estimates between the risky and the sure option. If β is 0, both options are equally likely to be chosen, irrespective of their expected values (reflecting a high degree of choice randomness, or an inability of the model to capture participants' choices). As β increases, the probability that the option with the highest expected value estimate will be chosen increases.

Parameter estimation

We applied all models to participants' trial-to-trial choice sequences, using a hierarchical Bayesian approach. This approach assumes that every participant has a different set of model parameters, which are drawn from group-level prior distributions [7]. The parameters governing the group-level prior distributions (hyperparameters) are also assigned prior distributions (hyperpriors). We estimated separate group-level parameters for each age group. As we are primarily interested in potential differences between the age groups, our primary variables of interest are the group-level means (i.e., the hyperparameters governing

the means of the group-level distributions). We denote the group-level mean of any parameter x as M^x.

Prior distributions. Group-level prior distributions for all parameters were assumed to be beta distributions [8]. Note that our choice to use beta prior distributions was unrelated to the assumption of the Bayesian ideal-observer models that outcome probabilities are represented as beta distributions. Beta distributions are typically defined by two shape parameters, but we reparametrized these in terms of a group-level mean and group-level precision [9]. The group-level means were assigned a uniform hyperprior on the interval [0,1] and the logarithms of the group-level precisions were assigned a uniform hyperprior on the interval [log(2), log(600)] [8]. Parameters for individual participants were drawn from the resulting group-level beta distributions.

As the beta distributions are restricted to the [0,1] interval, we transformed individual-level parameters with different ranges using linear transformations. Specifically, we transformed the range of Q_1 in all reinforcement-learning models to [0, 20] and the range of a_1 in all Bayesian ideal-observer models to [0, 2]; hence the initial expected value of the risky option could vary between 0 and 20 cents in all models. We transformed the range of utility parameter κ (Models 1C and 2C) to [0.5, 4], such that the subjective utility of a 20-cent outcome could vary between 10 and 80 cents. We transformed the ranges of update-rate parameter π (Model 2A, 2C, 2D) and positive and negative update-rate parameters π_+ and π_- (Model 2B) to [0, 10], such that the degree of updating could vary between no updating and strong initial updating. We transformed the range of uncertainty-bonus parameter φ (Model 2C) to [-50, 50], such that the effect of uncertainty on expected value could vary between highly positive and highly negative. Finally, we transformed the range of inverse-temperature parameter β to [0, 2], such that the probability of choosing the option with the higher expected value could vary from .5 (completely random) to higher than 0.99 (nearly deterministic).

MCMC sampling. We inferred posterior distributions for all model parameters using Markov chain Monte Carlo (MCMC) sampling, as implemented in JAGS [10] via the R2jags package [11]. We ran 3 independent MCMC chains and collected 40,000 posterior samples per chain. We discarded the first 20,000 iterations of each chain as burn-in. In addition, we only used every 5th iteration to remove autocorrelation. Consequently, we obtained 12,000 representative samples per parameter per model. All chains showed convergence (R-hat < 1.1) [12].

Supplemental Text 2: Latent mixture-model analysis

Our latent mixture model assumed that each participant's choice data is best explained by one out of several models, such that the total dataset reflects a mixture of these models. Specifically, in our mixture analyses we included two learning models, two gambler's fallacy models, and one simple choice model that does not consider the experienced outcomes. The two learning models were the models that best explained the adults' and adolescents' data in the previous analysis: the Bayesian ideal-observer model with two update rates and the reinforcement-learning model with nonlinear utility function, respectively. The two gambler's fallacy models were identical to these learning models, except that they use negative learning/update rates. Thus, the outcomes gambler's fallacy models assume that the value of the risky option decreases following each win outcome and increases following each no-win outcome, reflecting the incorrect belief that that occurred more frequent in the past will be less likely in the future. Finally, the last model—which we call the ε-risky model—has a fixed probability of choosing the risky option on each trial (determined by parameter ε); hence is insensitive to the experienced outcomes. This model can thus account for guessing behavior and for general tendencies to seek or avoid risk that are insensitive to outcome feedback.

Our mixture model analysis combines these five models within one "supermodel". Model-index parameter z—which can take five different values corresponding to each of the five models—controls which model is assigned to each participant [13]. The group-level distribution for z was assumed to be a probability distribution defined by a vector of five probabilities (which sum to 1), corresponding to the likelihoods for each of the five models included in the mixture model. These probabilities were in turn assigned a uniform Dirichlet hyperprior, such that each model was equally likely a priori. For each individual participant, this resulted in a multinomial posterior distribution for z, reflecting the number of iterations on which each model was assumed to account for the choice data. Thus, our mixture-model analysis infers which model is most plausible for each participant (the posterior mode of z), as well as the certainty of this model assignment (the proportion of samples that equal the posterior mode of z). We again estimated all model parameters in a hierarchical Bayesian way, using the same MCMC sampling method as in our other modeling analysis, and again estimated group-level parameters separately for each age group.

Supplemental Text 3: Regression analysis on risky choice behaviour including all participants assigned to a learning model by our mixture model analysis

We repeated the regression analysis on risky choice behaviour reported in the main text while including all participants who were assigned to a learning model by our mixture model analysis (28 adults, 25 mid-late adolescents and 18 early adolescents; Supplemental Figure 1). This analysis confimed that participants made more risky choices when the risky option had a higher expected value (main effect of block type: $z = 7.3$, $p < .001$). However, this analysis suggested that this effect emerged over trials in a linear way (block type x trial-linear interaction, $z = 6.7$, $p < .001$), as the block type x trial-quadratic interaction was no longer significant ($z = 1.2$, $p = .22$). Importantly, this analysis indicated that the speed at which participants optimized their choice behavior increased across the three age groups in a linear way, as reflected in block type x trial-linear x age group-linear ($z = 3.4$, $p < .001$) and block type x trial-quadratic x age group-linear ($z = 3.3$, $p = .005$) interactions, corroborating the results reported in the main text. Finally, the main effect of age group-linear was no longer significant ($z = 1.7$, $p = .09$), suggesting that the overall number of risky choices did not differ across the three age groups.

Supplemental Text 4: Model-recovery results

To validate our modeling results, we performed a model-recovery analysis using parameter estimates from fits of all models to the adult data and from fits of all models to the young adolescent data. Note that we used parameter estimates from the young adolescent group specifically because of their extremely low inverse temperature, potentially posing problems for model recovery. Specifically, we generated 20 datasets under each model, setting the group-level parameters (i.e., the group-level means and precisions) to the parameters obtained from model fits on the empirical data. Individual-level parameters were then drawn from the resulting group-level distributions and used to generate individual choice behaviour. Each generated dataset contained the same number of participants (i.e., n = 10 for the young adolescents and n = 19 for the adults) and number of trials (i.e., 10 blocks of 20 trials each per participant) as the empirical data.

Below we present confusion matrices, indicating the probability that data generated under a model is best fit by a certain model, and inversion matrices, indicating the probability that data best fit by a model is generated under a certain model14. These latter matrices can be used to validate our empirical results as we only know which model fitted the data best, not which generating process underlay the empirical data.

The empirical adult data were best fit by a Bayesian ideal-observer model with asymmetric update rates (Model 2B). Judging from the inversion matrix in the right hand panel of Supplemental Figure 5A, results indicate we can be 100% confident that the empirical data were indeed generated by a model from the Bayesian ideal-observer family, and 65% confident that the risk-sensitive mechanism underlying the empirical data was indeed asymmetric update rates. In 32% of cases, data were instead generated under an "uncertainty affects learning" model (Model 2D). In the remaining 3% of cases, data were instead generated without a risk-sensitive mechanisms (Model 2A).

The empirical adolescent data were best fit by a reinforcement-learning model with nonlinear utility function (Model 1C). Judging from the inversion matrix in the right hand panel of Supplemental Figure 5B, results indicate we can be 96% confident that the empirical data were indeed generated by a model from the reinforcement-learning family, and 80% confident that the risk-sensitive mechanism underlying the empirical data was indeed a nonlinear utility function. In the remaining 16% of cases, data were instead generated with a linear utility function (Model 1A).

Supplemental Text 5: Regression analysis on risky choice behavior in the adolescent sample using continuous age

To further explore how risky choice behavior developed over the course of adolescence, we performed a multilevel regression analysis similar to the one reported in the main text but in the adolescents only and with continuous age (Supplemental Figure 7) as independent variable. Similar to the results from the analysis including age group as independent variable, results from this analysis showed that adolescents made more risky choices when the risky option had a higher expected value (main effect of block type, $z = 10.8$, $p < .001$). This effect emerged over trials in a linear way (block type x trial-linear interaction, $z = 2.8$, $p = .005$) as opposed to a nonlinear way in the age-group analysis. Also, results showed that the speed at which adolescents optimized their choice behavior over trials tended to increase linearly with age (block type x trial-linear x age-linear interaction, $z = 1.8$, $p = .07$), although less clearly compared to the age-group results. Finally, the number of risky choices increased linearly with age (main effect of age-linear, $z = 2.1$, $p = .04$), again indicating within the adolescent sample that older participants made more risky choices.

Supplemental Text 6: Regression analyses including block effect.

We conducted an additional regression analysis to examine whether choice performance improved or deteriorated as the task progressed, for example due to practice effects or a limited attention span, and whether such potential time-on-task effects differed between our age groups. The dependent variable in this analysis was an index of the degree to which participants performed optimally—computed per block and participant—defined as the proportion of risky choices during the last 15 trials of each risk-advantageous block, and the proportion of sure choices during the last 15 trials of each risk-disadvantageous blocks. We excluded the first 5 trials of each block, during which participants were unlikely to know whether or not the risky option is advantageous. We also excluded the risk-neutral blocks from this analysis because there was no optimal choice strategy in these blocks. We then performed a multilevel analysis on this 'degree of optimality' variable, testing for effects of block, age group (linear and quadratic), and block x age group interactions. Results showed that the degree to which participants performed optimally did not change over the course of the task (main effect of block, $t(291) = .09$, $p = .93$), and did not differ across age groups (age-group linear, $t(239) = 1.2$, $p = .23$; age-group quadratic, $t(280) = .53$, $p = .60$). There were no block x age-group interactions either (both p's > .83).

In addition, to examine whether a potential block effect influenced the results from our main multilevel regression analysis, we included the effect of block in this analysis. This did not change the significance of any of the results reported in the main text.

Supplemental Figure 1. Mean proportion of choices for the risky option per trial, block type and age group, across all participants assigned to a learning model by our mixture-model analysis (compare to Figure 2 in the main text). This figure was created using RStudio 1.1.463, https://www.rstudio.com.

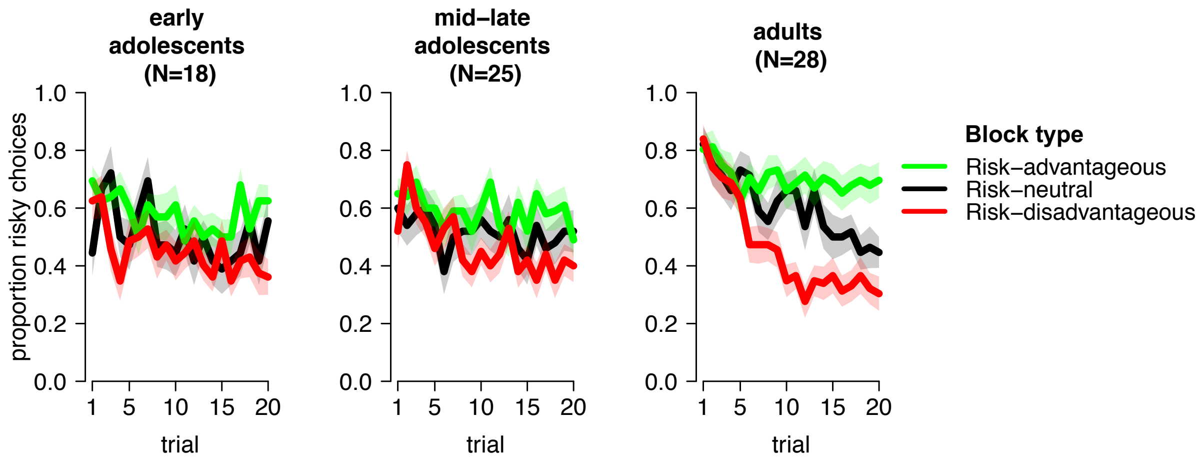

Supplemental Figure 2. Model fit. The average proportion of risky choices of the participants (data; solid lines) and the average model-predicted probability to choose the risky option (dotted lines) per trial, age group and block type. In each panel, green lines represent risk-advantageous blocks, black lines risk-neutral blocks, and red lines risk-disadvantageous blocks. The top row shows the model prediction for Model 1C (the best-fitting model for both groups of adolescents); the bottom row for model 2B (the best-fitting model for the adults). To obtain the model's predictions, we simulated choice data for each participant 20 times using the posterior medians of the individual-level parameters, and then plotted the average probability to choose the risky option. This figure was created using RStudio 1.1.463, https://www.rstudio.com.

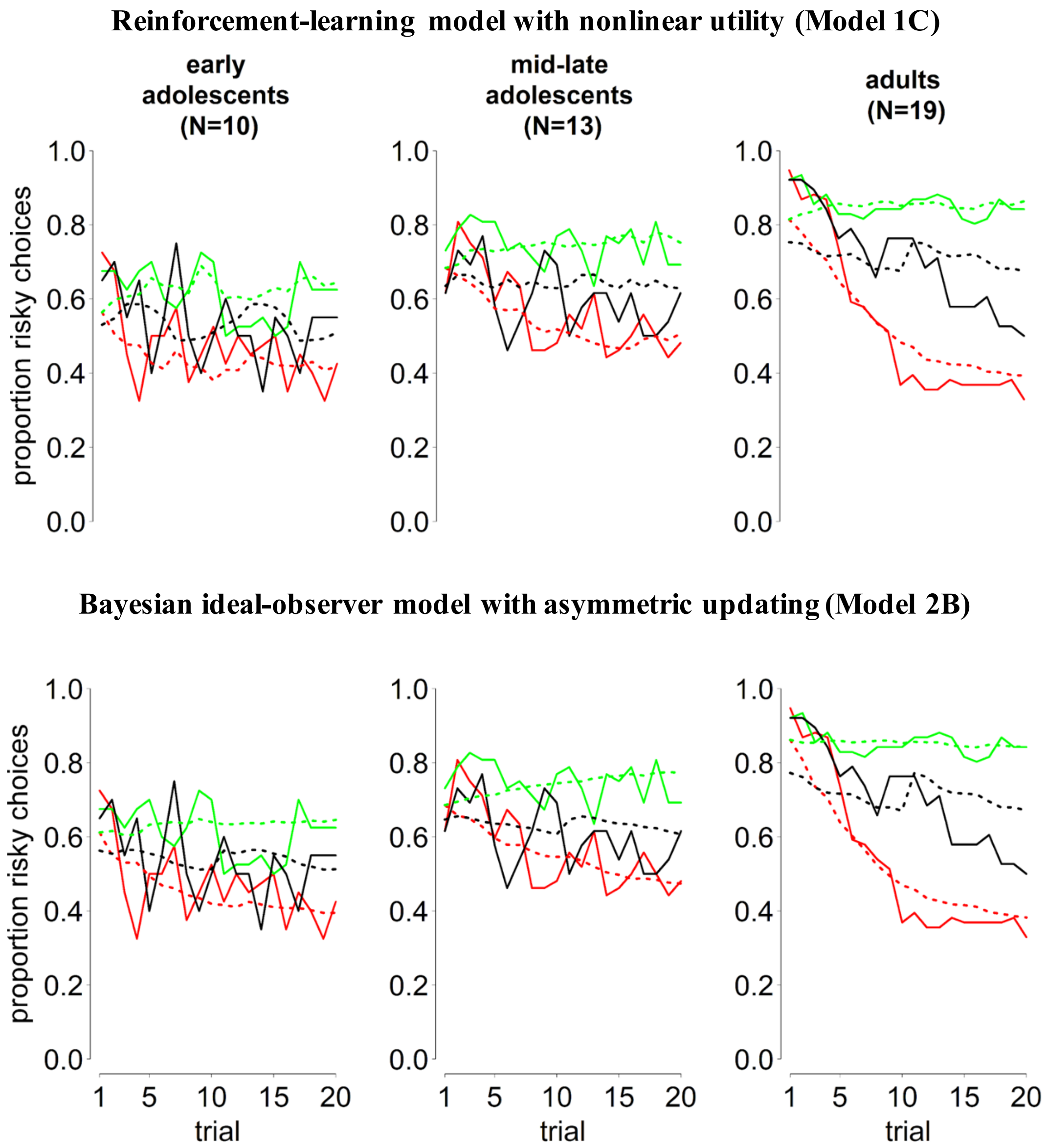

Supplemental Figure 3. Posterior distributions of the winning models' group-level mean parameters, per age group. Note that constant update rates in a beta binomial model (upper middle plot) produce a decrease in effective learning rate over trials, as shown in Fig. 4 in the main text. This figure was created using RStudio 1.1.463, https://www.rstudio.com.

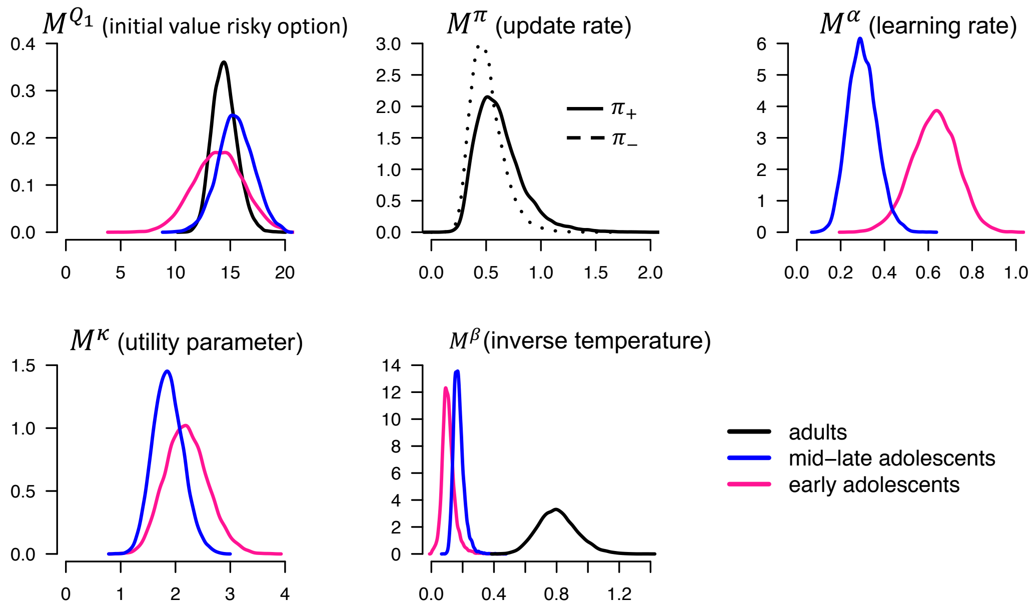

Supplemental Figure 4. Inferred strategy use for each individual participant according to our mixture model analysis. The black circles indicate the most plausible model for each participant, and the blue circles the less plausible models. The sizes of the circles represent the certainty that each participant's choices were best explained by each of the models (larger circles indicate higher certainty). This figure was created using RStudio 1.1.463, https://www.rstudio.com.

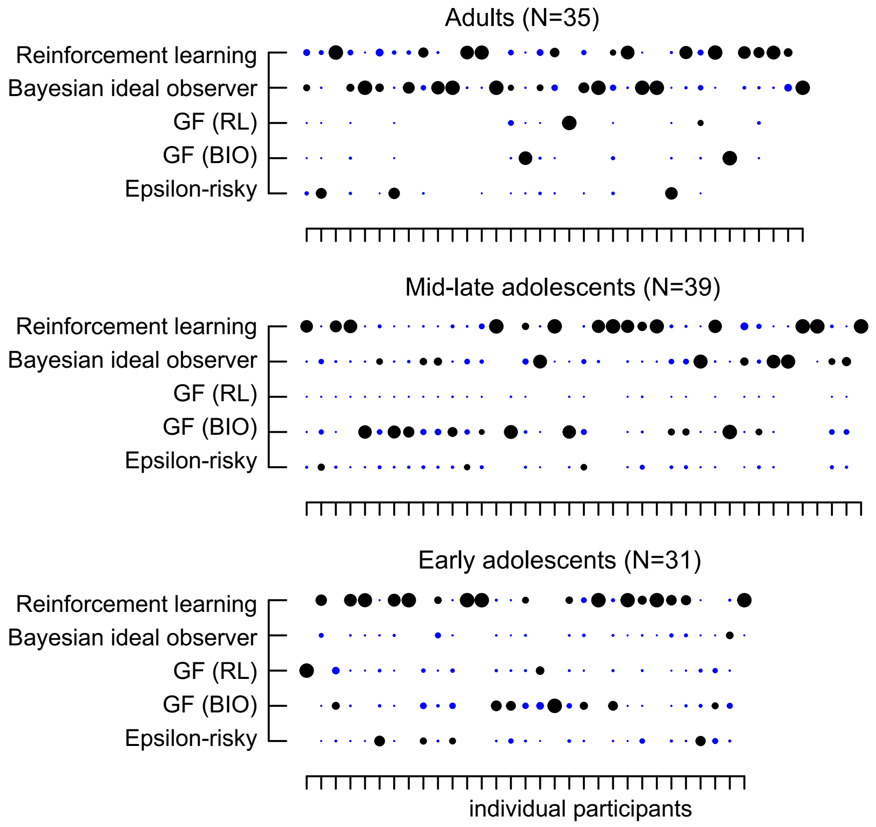

Supplemental Figure 5. Model-recovery results for the adults (A) and young adolescents (B). Models 1-3 represent reinforcement-learning, Bayesian ideal-observer, and epsilon-risky models, respectively. Accompanying letters A-D indicate basic, asymmetric learning, nonlinear utility, and “uncertainty affects learning” models, respectively. The confusion matrix indicates the probability that data generated under a model is best fit by a certain model, and the inversion matrix indicates the probability that data best fit by a model is generated under a certain model. Green outlines indicate the best-fitting model for the adult (A) and young adolescent (B) data. This figure was created using RStudio 1.1.463, https://www.rstudio.com.

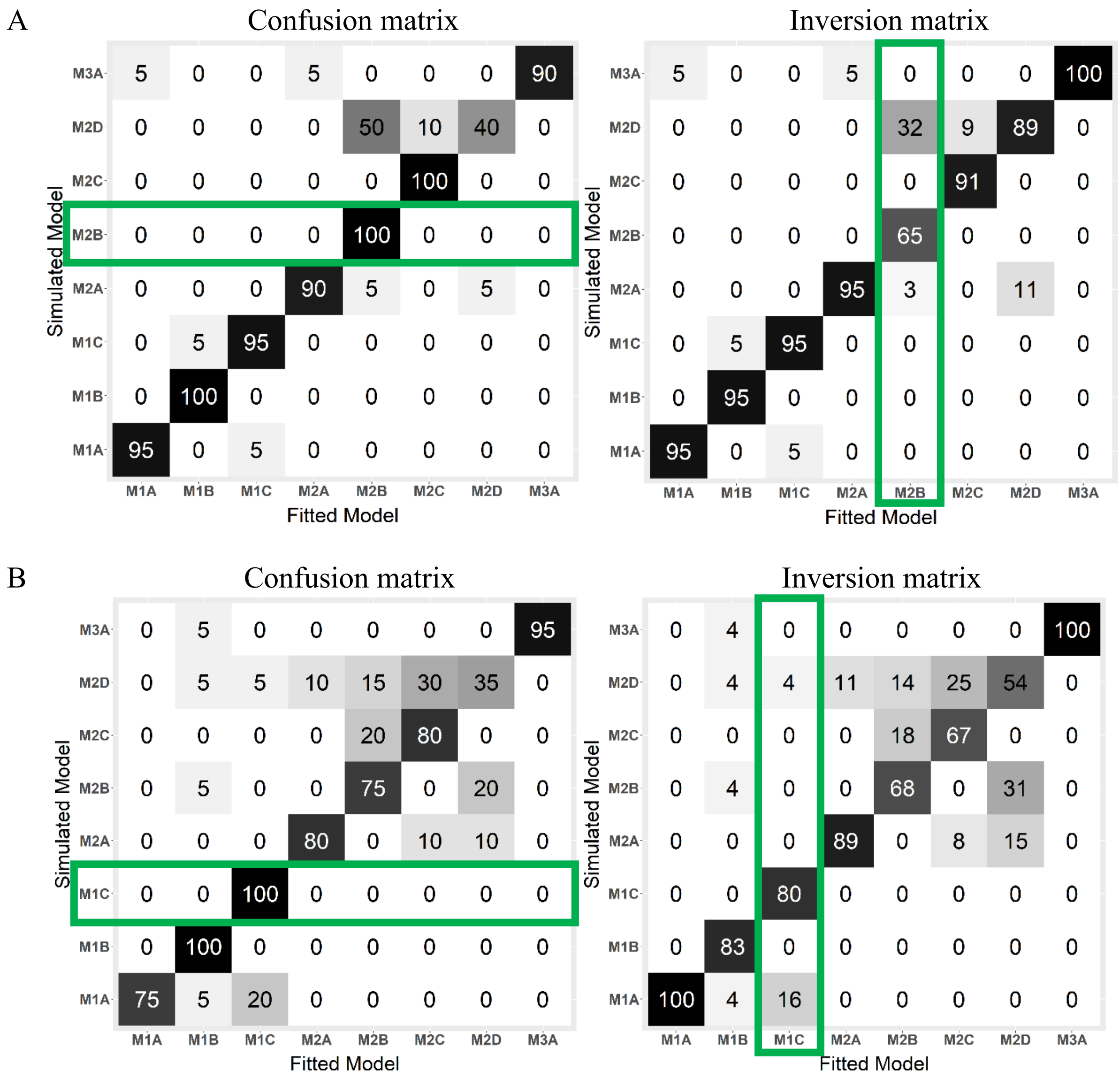

Supplemental Figure 6. Comparison of models that do vs. do not re-initialize the value of the risky option at the beginning of each new block. A possible alternative explanation for adolescents' poor learning performance is that they understood and treated each block as a new learning context. To test this hypothesis, we recoded the best-fitting models in each age group such that the risky choice value was not re-initialized at the beginning of each block. ΔDIC values reflect the difference in model fit between the models with and without re-initialized values of the risky option. A positive ΔDIC value indicates that the model with re-initialized values has a better fit (lower DIC). Results show that models with re-initialized risky values fitted the data better in all age groups, suggesting that participants treated each block as a new learning context. This figure was created using RStudio 1.1.463, https://www.rstudio.com.

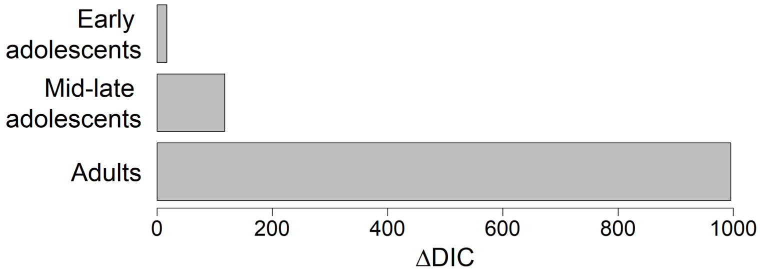

Supplemental Figure 7. Age distribution in adolescents. This figure was created using RStudio 1.1.463, https://www.rstudio.com.

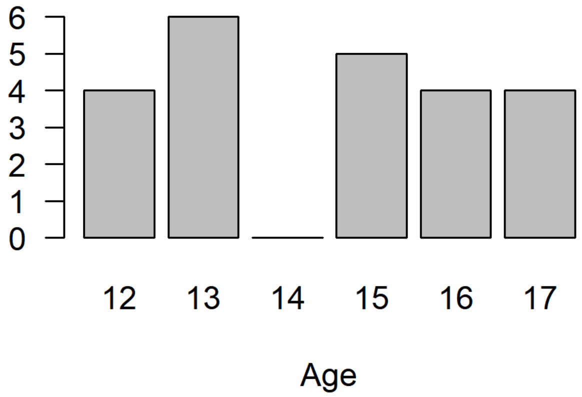

Supplemental References

1 Rescorla, R. A. & Wagner, A. R. in Classical conditioning II: current research and theory (eds Abraham H. Black & William F. Prokasy) 64-99 (Appleton-Century-Crofts, 1972).

2 van den Bos, W., Cohen, M. X., Kahnt, T. & Crone, E. A. Striatum-medial prefrontal cortex connectivity predicts developmental changes in reinforcement learning. Cereb Cortex **22**, 1247-1255, doi:10.1093/cercor/bhr198 (2012).

3 Niv, Y., Edlund, J. A., Dayan, P. & O'Doherty, J. P. Neural prediction errors reveal a risk-sensitive reinforcement-learning process in the human brain. J Neurosci **32**, 551-562, doi:10.1523/JNEUROSCI.5498-10.2012 (2012).

4 Bernoulli, D. Exposition of a new theory on the measurement of risk. Econometrica **22**, 23-36 (1954).

5 Kakade, S. & Dayan, P. Dopamine: generalization and bonuses. Neural Netw **15**, 549-559, doi:10.1016/s0893-6080(02)00048-5 (2002).

6 Daw, N. D., O'Doherty, J. P., Dayan, P., Seymour, B. & Dolan, R. J. Cortical substrates for exploratory decisions in humans. Nature **441**, 876-879, doi:10.1038/nature04766 (2006).

7 Gelman, A. Bayesian data analysis. Third edition. (CRC Press, 2014).

8 Steingroever, H., Wetzels, R. & Wagenmakers, E. J. Absolute performance of reinforcement-learning models for the Iowa Gambling Task. Decision **1** (2014).

9 Smithson, M. & Verkuilen, J. A better lemon squeezer? Maximum-likelihood regression with beta-distributed dependent variables. Psychol Methods **11**, 54-71, doi:10.1037/1082-989X.11.1.54 (2006).

10 Plummer, M. JAGS: a program for analysis of bayesian graphical models using gibbs sampling. Proceedings of the 3rd International Workshop on Distributed Statistical Computing 124:125 (2003).

11 Su, Y.-S. & Yajima, M. R2jags: Using R to run “JAGS.”. R Packages, doi:https://doi.org/http://cran.r-project.org/package=R2jags (2015).

12 Gelman, A. & Rubin, D. B. Inference from Iterative Simulation Using Multiple Sequences. Statistical Science **7**, 457-472 (1992).

13 Lodewyckx, T. et al. A tutorial on Bayes factor estimation with the product space method. Journal of Mathematical Psychology **55** (2011).

14 Wilson, R. C. & Collins, A. G. Ten simple rules for the computational modeling of behavioral data. Elife **8**, doi:10.7554/eLife.49547 (2019).
